# Systematic review of guidelines for internal validity in the design, conduct and analysis of preclinical biomedical experiments involving laboratory animals

**DOI:** 10.1136/bmjos-2019-100046

**Published:** 2020-04-15

**Authors:** Jan Vollert, Esther Schenker, Malcolm Macleod, Anton Bespalov, Hanno Wuerbel, Martin Michel, Ulrich Dirnagl, Heidrun Potschka, Ann-Marie Waldron, Kimberley Wever, Thomas Steckler, Tom van de Casteele, Bruce Altevogt, Annesha Sil, Andrew S C Rice, The EQIPD WP3 study group members, Jan Vollert

**Affiliations:** 1 Pain Medicine, Department of Surgery and Cancer, Faculty of Medicine, Imperial College London, London, UK; 2 Institut de Recherches Internationales Servier, Suresnes, Île-de-France, France; 3 Centre for Clinical Brain Sciences, Edinburgh Medical School, The University of Edinburgh, Edinburgh, Scotland, UK; 4 Partnership for Assessment and Accreditation of Scientific Practice, Heidelberg, Germany; 5 Valdman Institute of Pharmacology, Pavlov First State Medical University of Saint Petersburg, Sankt Petersburg, Russian Federation; 6 Division of Animal Welfare, Vetsuisse Faculty, VPH Institute, University of Bern, Bern, Switzerland; 7 Universitätsmedizin Mainz, Johannes Gutenberg Universität Mainz, Mainz, Rheinland-Pfalz, Germany; 8 Department of Experimental Neurology, Charité–Universitätsmedizin Berlin, Berlin, Germany; 9 Institute of Pharmacology, Toxicology, and Pharmacy, Ludwig-Maximilians-Universitat Munchen, Munchen, Bayern, Germany; 10 Systematic Review Centre for Laboratory Animal Experimentation, Department for Health Evidence, Nijmegen Institute for Health Sciences, Radboud Universiteit, Nijmegen, Gelderland, Netherlands; 11 Janssen Pharmaceutica, Beerse, Antwerpen, Belgium; 12 Pfizer, New York, New York, USA; 13 Institute of Medical Sciences, University of Aberdeen, Aberdeen, UK

**Keywords:** scientific rigor, bias, internal validity, preclinical studies, animal studies

## Abstract

Over the last two decades, awareness of the negative repercussions of flaws in the planning, conduct and reporting of preclinical research involving experimental animals has been growing. Several initiatives have set out to increase transparency and internal validity of preclinical studies, mostly publishing expert consensus and experience. While many of the points raised in these various guidelines are identical or similar, they differ in detail and rigour. Most of them focus on reporting, only few of them cover the planning and conduct of studies. The aim of this systematic review is to identify existing experimental design, conduct, analysis and reporting guidelines relating to preclinical animal research. A systematic search in PubMed, Embase and Web of Science retrieved 13 863 unique results. After screening these on title and abstract, 613 papers entered the full-text assessment stage, from which 60 papers were retained. From these, we extracted unique 58 recommendations on the planning, conduct and reporting of preclinical animal studies. Sample size calculations, adequate statistical methods, concealed and randomised allocation of animals to treatment, blinded outcome assessment and recording of animal flow through the experiment were recommended in more than half of the publications. While we consider these recommendations to be valuable, there is a striking lack of experimental evidence on their importance and relative effect on experiments and effect sizes.

## Introduction

In recent years, there has been growing awareness of the negative repercussions of shortcomings in the planning, conduct and reporting of preclinical animal research.^1 2^ Several initiatives involving academic groups, publishers and others have set out to increase the internal validity and reliability of primary research studies and the resulting publications. Additionally, several experts or groups of experts across the biomedical spectrum have published experience and opinion-based guidelines and guidance. While many of the points raised are broadly similar between these various guidelines (probably in part reflecting the observation that many experts in the field are part of more than one initiative), they differ in detail, rigour and, in particular, whether they are broadly generalisable or specific to a single field. While all these guidelines cover the reporting of experiments, only a few specifically address rigorous planning and conduct of studies,^3 4^ which might increase validity from the earliest possible point.^5^ Consequently, it is difficult for researchers to choose which guidelines to follow, especially at the stage of planning future studies.

We aimed to identify all existing guidelines and reporting standards relating to experimental design, conduct and analysis of preclinical animal research. We also sought to identify literature describing (either through primary research or systematic review) the prevalence and impact of perceived risks of bias pertaining to the design, conduct and analysis and reporting of preclinical biomedical research. While we focus on internal validity as influenced by experimental design, conduct and analysis we recognise that factors such as animal housing and welfare are highly relevant to the reproducibility and generalisability of experimental findings; however, these factors are not considered in this systematic review.

## Methods

The protocol for this systematic review has been published in ref 6. The following amendments to the systematic review protocol were made: in addition to the systematic literature search, to capture standards set by funders or organisations that are not (or not yet) published, it was planned to conduct a Google search for guidelines published on the websites of major funders and professional organisations using the systematic search string below.^6^ This search, however, yielded either no returns, or, in the case of the National Institute of Health, identified over 193 000 results, which was an unfeasibly large number to screen. Therefore, for practical reasons this part of the search was excluded from the initial search strategy. Reassessing the goals of this review, we decided to focus on internal validity, in the protocol we used the term ‘internal validity and reproducibility’. In the protocol, we mention that the aim of this systematic review is an effort to harmonise guidelines and create a unified framework. This is still under way and will be published separately.

### Search strategy

We systematically searched PubMed, Embase via Ovid and Web of Science to identify the guidelines published in English language in peer-reviewed journals before 10 January 2018 (the day the search was conducted), using appropriate terms for each database optimised from the following search string (as can be found in the protocol^6^):

(guideline OR recommendation OR recommendations) AND (‘preclinical model’ OR ‘preclinical models’ OR ‘disease model’ OR ‘disease models’ OR ‘animal model’ OR ‘animal models’ OR ‘experimental model’ OR ‘experimental models’ OR ‘preclinical study’ OR ‘preclinical studies’ OR ‘animal study’ OR ‘animal studies’ OR ‘experimental study’ OR ‘experimental studies’).^6^

Furthermore, as many of the researchers participating in the European Quality in Preclinical Data project (http://eqipd.org/) are experts in the field of experimental standardisation, they were contacted personally to identify additional relevant publications.

### Inclusion and exclusion criteria

We included all articles or systematic reviews in English which described or reviewed guidelines making recommendations intended to improve the validity or reliability (or both) of preclinical animal studies through optimising their design, conduct and analysis. Articles that focused on toxicity studies or veterinary drug testing were not included. Although reporting standards were not the key primary objective of this systematic review these were also included, as they might contain useful relevant information.

### Screening and data management

We combined the search results from all sources and identified duplicate search returns and the publication of identical guidelines by the same author group in several based on the PubMed ID, DOI, and the title, journal and author list. Unique references were then screened in two phases: (1) screening for eligibility based on title and abstract, followed by (2) screening for definitive inclusion based on full text. Screening was performed using the Systematic Review Facility (SyRF) platform (http://syrf.org.uk). Ten reviewers contributed to the screening phase; each citation was presented to two independent reviewers with a real-time computer-generated random selection of the next citation to be reviewed. Citations remained available for screening until two reviewers agreed that it should be included or excluded. If the first two reviewers had disagreed the citation was offered to a third, but reviewers were not aware of previous screening decisions. A citation could not be offered to the same reviewer twice. Reviewers were not blinded to the authors of the presented record. In the first stage, two authors screened the title and abstract of the retrieved records for eligibility based on predefined inclusion criteria (see above). The title/abstract screening stage aimed to maximise sensitivity rather than specificity—any paper considered to be of any possible interest was included.

Articles included after the title-abstract screening were retrieved as full texts. Articles for which no full-text version could be obtained were excluded from the review. Full texts were then screened for definite inclusion and data extraction. At both screening stages, disagreements between reviewers were resolved by additional screening of the reference by a third adjudicating reviewer, who was unaware of the individual judgements of the first two reviewers. All data were stored on the SyRF platform.

### Extraction, aggregation and diligence classification

From the publications identified, we extracted recommendations on the planning, conduct and reporting of preclinical animal studies as follows:

Elements of the included guidelines were identified using an extraction form (box 1) inspired by the results from Henderson *et al*.^5^ Across guidelines, the elements were ranked based on the number of guidelines in which that element appeared. Extraction was not done in duplicate, but only once. As the extracted results in this case are not quantitative, but qualitative, meta-analysis and risk of bias assessment are not appropriate for this review. Still, we applied a diligence classification of the guidelines based on the following system, improving level of evidence from 1 to 3 and support from A to B:

Box 1 **Extraction form**

- Matching or balancing treatment allocation of animals.
- Matching or balancing sex of animals across groups.
- Standardised handling of animals.
- Randomised allocation of animals to treatment.
- Randomisation for analysis.
- Randomised distribution of animals in the animal facilities.
- Monitoring emergence of confounding characteristics in animals.
- Specification of unit of analysis.
- Addressing confounds associated with anaesthesia or analgesia.
- Selection of appropriate control groups.
- Concealed allocation of treatment.
- Study of dose–response relationships.
- Use of multiple time points measuring outcomes.
- Consistency of outcome measurement.
- Blinding of outcome assessment.
- Establishment of primary and secondary end points.
- Precision of effect size.
- Management of conflicts of interest.
- Choice of statistical methods for inferential analysis.
- Recording of the flow of animals through the experiment.
- A priori statements of hypothesis.
- Choice of sample size.
- Addressing confounds associated with treatment.
- Characterisation of animal properties at baseline.
- Optimisation of complex treatment parameters.
- Faithful delivery of intended treatment.
- Degree of characterisation and validity of outcome.
- Treatment response along mechanistic pathway.
- Assessment of multiple manifestations of disease phenotype.
- Assessment of outcome at late/relevant time points.
- Addressing treatment interactions with clinically relevant comorbidities.
- Use of validated assay for molecular pathways assessment.
- Definition of outcome measurement criteria.
- Comparability of control group characteristics to those of previous studies.
- Reporting on breeding scheme.
- Reporting on genetic background.
- Replication in different models of the same disease.
- Replication in different species or strains.
- Replication at different ages.
- Replication at different levels of disease severity.
- Replication using variations in treatment.
- Independent replication.
- Addressing confounds associated with experimental setting.
- Addressing confounds associated with setting.
- Preregistration of study protocol and analysis procedures.
- Pharmacokinetics to support treatment decisions.
- Definition of treatment.
- Interstudy standardisation of end point choice.
- Define programmatic purpose of research.
- Interstudy standardisation of experimental design.
- Research within multicentre consortia.
- Critical appraisal of literature or systematic review during design phase.
- (Multiple) free text.

1. Recommendations of individuals or small groups of individuals based on individual experience only.

- Published stand-alone.
- Endorsed or initiated by at least one publisher or scientific society as stated in the publication.

2. Recommendations by groups of individuals, through a method which included a Delphi process or other means of structured decision-making.

- Published stand-alone.
- Endorsed or initiated by at least one publisher or scientific society as stated in the publication.

3. Recommendations based on a systematic review.

- Published stand-alone.
- Endorsed or initiated by at least one publisher or scientific society as stated in the publication.

## Results

### Search and study selection

**Figure 1:**
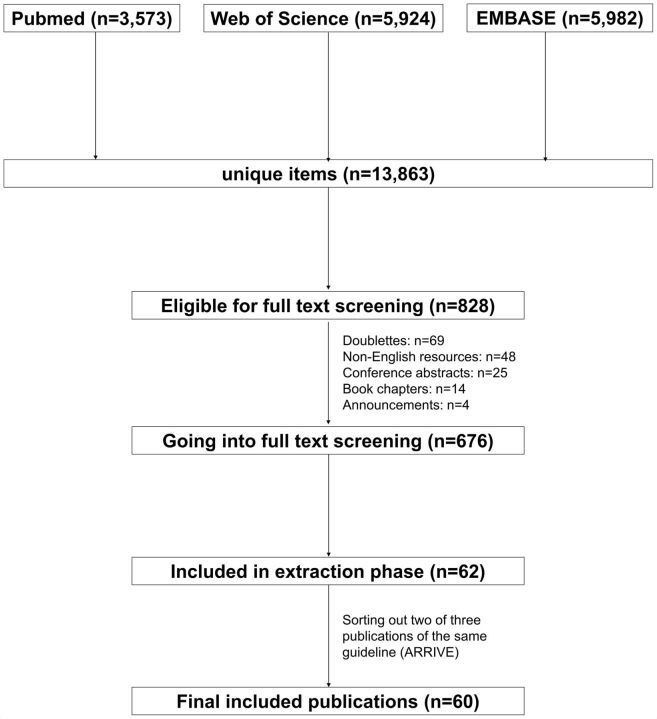
Search flow chart. ARRIVE, Animal Research: Reporting of In Vivo Experiments.

A flow chart of the search results and screening process is found in figure 1. Our systematic search returned 13 863 results, with 3573 papers from PubMed, 5924 from Web of Science and 5982 from Embase. After first screening on title and abstract, 828 records were eligible for the full-text screening stage. After removing duplications (69), non-English resources (48), conference abstracts (25), book chapters (14) and announcements (4), 676 records remained. Of these, 62 publications were retained after full-text screening. We later identified two further duplicate publications of the same guidelines in different journals, giving a final list of 60 publications.^5 7–65^

The project members did not identify any additional papers that had not been identified by the systematic search.

### Diligence classification

More than half of the included publications (32) were narrative reviews that fell under the 1A category of our rating system (recommendations of individuals or small groups of individuals based on individual experience only, published stand-alone).^7 9 10 14 15 18 20 25 27 29 30 33 35 36 39 41–43 45 47–55 57 60 61 65^ An additional 22 publications were consensus papers or proceedings of consensus meetings for journals or scientific or governmental organisations (category 1B).^3 4 8 12 13 17 19 24 26 28 32 34 37 38 44 46 56 59 62–64 66^ None of these reported the use of a Delphi process or systematic review of existing guidelines. The remaining six publications were systematic reviews of the literature (category 3A).^5 11 21 31 40 58^

### Extracting components of published guidance

From the 60 publications finally included, we extracted 58 unique recommendations on the planning, conduct and reporting of preclinical animal studies. The absolute and relative frequency for each of the extracted recommendations is provided in table 1. Sample size calculations, adequate statistical methods, concealed and randomised allocation of animals to treatment, blinded outcome assessment and recording of animal flow through the experiment were recommended in more than half of the publications. Only a few publications (≤5) mentioned preregistration of experimental protocols, research conducted in large consortia, replication at different levels of disease or by variation in treatment and optimisation of complex treatment parameters. The extraction form allowed the reviewers in free-text fields to identify and extract additional recommendations not covered in the prespecified list, but this facility was rarely used, with only ‘publication of negative results’ and ‘clear specification of exclusion criteria’ extracted in this way by more than one reviewer. The full results table of this stage is published as csv file on figshare under the DOI 10.6084/m9.figshare.9815753.

**Table 1:** Extraction results

Recommendation	Absolute frequency	Relative frequency (%)
Adequate choice of sample size	41	68
Blinding of outcome assessment	41	68
Choice of statistical methods for inferential analysis	38	63
Randomised allocation of animals to treatment	38	63
Concealed allocation of treatment	31	52
Recording of the flow of animals through the experiment	31	52
A priori statements of hypothesis	30	50
Selection of appropriate control groups	29	48
Characterisation of animal properties at baseline	28	47
Addressing confounds associated with setting	23	38
Definition of outcome measurement criteria	23	38
Reporting on genetic background	23	38
Matching or balancing sex of animals across groups	20	33
Degree of characterisation and validity of outcome	19	32
Consistency of outcome measurement	18	30
Monitoring emergence of confounding characteristics in animals	18	30
Precision of effect size	18	30
Study of dose–response relationships	18	30
Addressing confounds associated with experimental setting	17	28
Establishment of primary and secondary end points	17	28
Reporting on breeding scheme	16	27
Assessment of outcome at late/relevant time points	15	25
Independent replication	15	25
Matching or balancing treatment allocation of animals	15	25
Specification of unit of analysis	15	25
Randomisation for analysis	14	23
Replication in different species or strains	14	23
Standardised handling of animals	14	23
Addressing confounds associated with anaesthesia or analgesia	13	22
Replication in different models of the same disease	13	22
Addressing confounds associated with treatment	12	20
Management of conflicts of interest	11	18
Treatment response along mechanistic pathway	11	18
Interstudy standardisation of experimental design	10	17
Assessment of multiple manifestations of disease phenotype	9	15
Use of multiple time points measuring outcomes	9	15
Definition of treatment	8	13
Interstudy standardisation of end point choice	8	13
Pharmacokinetics to support treatment decisions	8	13
Randomised distribution of animals in the animal facilities	8	13
Use of validated assay for molecular pathways assessment	8	13
Faithful delivery of intended treatment	7	12
Addressing treatment interactions with clinically relevant comorbidities	6	10
Any additional elements that do not fit in the list above	6	10
Comparability of control group characteristics to those of previous studies	6	10
Critical appraisal of literature or systematic review during design phrase	6	10
Define programmatic purpose of research	6	10
Replication at different ages	6	10
Replication using variations in treatment	5	8
Optimisation of complex treatment parameters	4	7
Replication at different levels of disease severity	4	7
Research within multicentre consortia	4	7
Preregistration of study protocol and analysis procedures	3	5

## Discussion

Based on our systematic literature search and screening using predefined inclusion and exclusion criteria, we identified 60 published guidelines for the planning, conduct or reporting of preclinical animal research. From these publications, we extracted a comprehensive list of 58 experimental rigour recommendations that the authors had proposed as being important to increase the internal validity of animal experiments. Most recommendations were repeated in a relevant proportion of the publications (sample size calculations, adequate statistical methods, concealed and randomised allocation of animals to treatment, blinded outcome assessment and recording of animal flow through the experiment in more than half of the cases), showing that there is at least some consensus for those recommendations. In many cases this may be because authors are on more than one of the expert committees for these guidelines, and many of them build on the same principles and cite the same sources of inspiration (ie, doing for the field what the Consolidated Standards of Reporting Trials did for clinical trials).^66 67^ There are also reasons why the consensus was not universal—many of the publications focus on single aspects (eg, statistics^21^ or sex differences^60^ or specific medical fields or diseases).^13 37 38 63^ In addition, the narrative review character of many of the publications may have led to authors focusing on elements they considered more important than others.

Indeed, more than half (32 out of 60) of the publications reviewed here were topical reviews by a small group of authors (usually fewer than five). Another 22 (37%) were proceedings of consensus meetings or consensus papers set in motion by professional scientific or governmental organisations. It is noteworthy that none of these publications provide any rationale or justification for the validity of their recommendations. None used a Delphi process or other means of structured decision-making as suggested for clinical guidelines^68^ to reduce bias,^69^ and none reported using a systematic review of existing guidelines to inform themselves about literature. Of course, many of these expert groups will have been informed by pre-existing reviews (the remaining six included here were systematic literature reviews). However, there is a consistent feature across recommendations—that the steps recommended to increase validity are considered to be self-evident, and a basis in experiments and evidence is seldom linked or provided. There are hints that applying these principles does contribute to internal validity, as it has been shown that the reporting of measures to reduce risks of bias is associated with smaller outcome effect sizes,^70^ while other studies have not found such.^71^ However, it is unclear if these measures taken are the perfect ones to reduce bias, or if they are merely surrogate markers for more awareness and thus more thorough research conduct. We consider this to be problematic for at least two reasons: first, to increase compliance with guidelines it is crucial to keep them as simple and as easy to implement as possible. An endless checklist can easily lead to fatalistic thinking in researchers desperately wanting to publish, and it could be debated whether guidelines are seen by some researchers as hindering their progression rather than being an aide to conducting the best possible science, still, there is a difference between an ‘endless’ list and a ‘minimal set of rules’ that guarantees good research reproducibility. Second, each procedure that is added to experimental set-up can in itself lead to sources of variation, so these should be minimised unless it can be shown that they add value to experiments.

Compliance is a significant problem for guidelines, as recently reported with the widely adopted Animal Research: Reporting of In Vivo Experiments (ARRIVE) guidelines of the UK’s National Centre for the 3Rs.^66 72^ This is not attributed to blind spots in the ARRIVE guidelines. While enforcement by endorsing journals may be important,^73 74^ a recent randomised blinded controlled study suggests that even an insistence of completing an ARRIVE checklist has little or no impact on reporting quality.^75^ We believe that training and availability of tools to improve research quality will facilitate implementation of guidelines over time, as they become more prominent in researchers’ mindset.

This systematic review has important limitations. The main limitation is that we used single extraction only, which was due to feasibility, but creates a source of uncertainty that we cannot rule out. We decided so as we think the bias created here is significantly lower than in a quantitative extraction that includes meta-analysis. Protocol-wise, we only included publications in English language, reflecting the limited language pool of our team. Our broad search strategy identified more than 13 000 results, but we did not identify reports or systematic reviews of primary research showing the importance of specific recommendations,^76^ which must reflect a weakness in our search strategy. Additionally, our plan to search the websites of professional organisations and funding bodies failed due to reasons of practicality. Limiting the results included from a Google search would have been a practical solution to overcome this issue, which we failed to decide at protocol generation. Although being aware of single recommendations outside of publication, we did not include those to keep methods reproducible. In addition, we focused the search on ‘guidelines’, instead of a broader focus on adding, for example, ‘guidance’, ‘standard’ or ‘policy’, as we feared these terms would inflate the search results by magnitude (particularly ‘standard’ is a broadly used word). Hence, we cannot ascertain whether we have included all important sources of literature. As hinted above, the results presented here also only paint an overview of the literature consensus, which should by no means be mistaken for an absolute ground truth of which steps need to be taken to improve internal validity in animal experiments. Indeed, literature debating the quality of these measures is sparse, and many of them have been borrowed from the clinical trials community or been considered self-evident from the literature. There is an urgent need for experimental testing of the importance of most of these measures, to provide better evidence of their effect.
